# Surgery for Hip Fracture Yields Societal Benefits That Exceed the Direct Medical Costs

**DOI:** 10.1007/s11999-014-3820-6

**Published:** 2014-08-05

**Authors:** Qian Gu, Lane Koenig, Richard C. Mather, John Tongue

**Affiliations:** 1 Econometrica, Inc, Bethesda, MD USA; 2 KNG Health Consulting LLC, 15245 Research Blvd, Suite 305, Rockville, MD 20850 USA; 3 Duke Orthopaedic Surgery, Durham, NC USA; 4 Oregon Health and Science University, Tualatin, OR USA

## Abstract

**Background:**

A hip fracture is a debilitating condition that consumes significant resources in the United States. Surgical treatment of hip fractures can achieve better survival and functional outcomes than nonoperative treatment, but less is known about its economic benefits.

**Questions/purposes:**

We asked: (1) Are the societal benefits of hip fracture surgery enough to offset the direct medical costs? (2) Nationally, what are the total lifetime benefits of hip fracture surgery for a cohort of patients and to whom do these benefits accrue?

**Methods:**

We estimated the effects of surgical treatment for displaced hip fractures through a Markov cohort analysis of patients 65 years and older. Assumptions were obtained from a systematic literature review, analysis of Medicare claims data, and clinical experts. We conducted a series sensitivity analyses to assess the effect of uncertainty in model parameters on our estimates. We compared costs for medical care, home modification, and long-term nursing home use for surgical and nonoperative treatment of hip fractures to estimate total societal savings.

**Results:**

Estimated average lifetime societal benefits per patient exceeded the direct medical costs of hip fracture surgery by USD 65,000 to USD 68,000 for displaced hip fractures. With the exception of the assumption of nursing home use, the sensitivity analyses show that surgery produces positive net societal savings with significant deviations of 50% from the base model assumptions. For an 80-year-old patient, the breakeven point for the assumption on the percent of patients with hip fractures who would require long-term nursing home use with nonoperative treatment is 37% to 39%, compared with 24% for surgical patients. Nationally, we estimate that hip fracture surgery for the cohort of patients in 2009 yields lifetime societal savings of USD 16 billion in our base model, with benefits and direct costs of USD 21 billion and USD 5 billion, respectively. For an 80-year-old, societal benefits ranged from USD 2 billion to USD 32 billion, using our range of estimates for nursing home use among nonoperatively treated patients who are immobile after the fracture.

**Conclusions:**

Surgical treatment of hip fractures produces societal savings. Although the magnitude of these savings depends on model assumptions, the finding of societal savings is robust to a range of parameter values.

**Level of Evidence:**

Level III, economic and decision analyses. See the Instructions for Authors for a complete description of levels of evidence.

**Electronic supplementary material:**

The online version of this article (doi:10.1007/s11999-014-3820-6) contains supplementary material, which is available to authorized users.

## Introduction

More than 300,000 patients sustain a hip fracture each year in the United States [3]. These serious injuries often result in nursing home stays, increased mortality, and overall lower quality of life [5, 13, 20, 24, 25, 41]. Hip fracture rates increase exponentially with age [34], with approximately 90% of hip fractures occurring in people older than 65 years [36]. As the US population ages, the incidence of hip fracture is expected to increase substantially. One study estimated that by 2040, the annual incidence of hip fractures will exceed 500,000 in the United States population [32]. These continuing trends will place a financial burden on patients, families, insurers, and governments.

Although surgery is the predominant treatment strategy for hip fractures because it reduces mortality risk and improves physical functioning for many patients, less is known about its societal cost implications. With policy makers and payers increasingly focused on value, it is critical to understand the return from healthcare spending. In this study, we considered the economic returns from spending on hip fracture. The broader economic effects of surgical treatment for hip fractures are reflected in direct medical costs and indirect costs, including long-term medical costs, custodian costs associated with nursing home stays, and the cost of home modification. Estimates of the societal benefits of medical treatment help establish a larger context for critically viewing overall healthcare spending, which informs health policy decision making and healthcare resource allocation.

We evaluated the economic value of surgical treatment of hip fractures for patients who undergo surgery and society in general by addressing two questions: (1) Are the societal benefits of hip fracture surgery enough to offset the direct medical costs? (2) Nationally, what are the total lifetime benefits of hip fracture surgery for a cohort of patients and to whom do these benefits accrue?

## Materials and Methods

**Fig. 1A–B:**
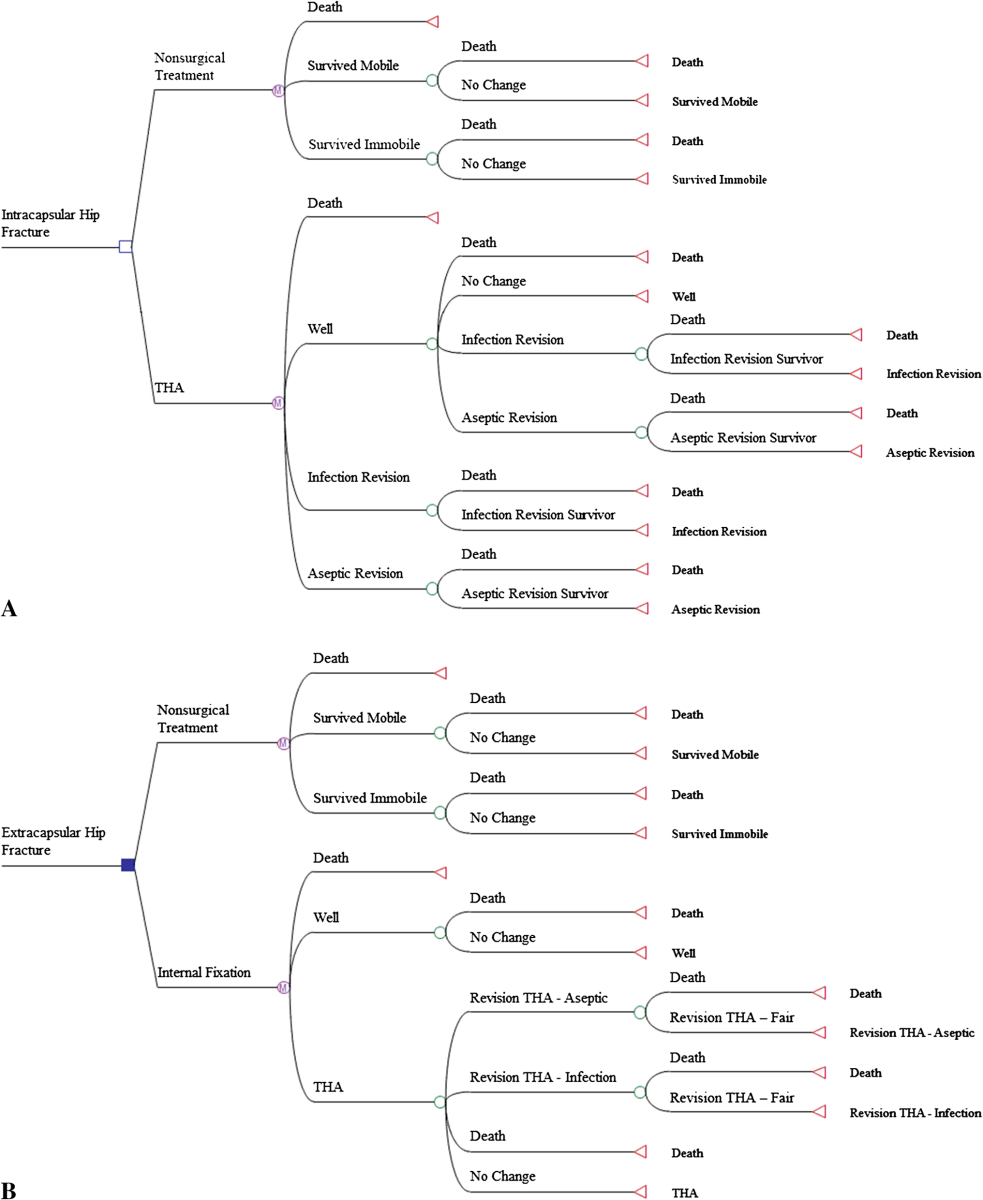
(**A**) A decision tree shows the treatment pathway and health states in the Markov model of hip fractures. The surgical branch of intracapsular fractures consists of four health states: dead, well, infection revision, and aseptic revision (infection and aseptic revisions are represented by one oval in the figure). In the first year after surgery, living patients enter the well state. The well state includes good and fair outcomes. For patients in the well state, they can die, stay in that health state, or have a revision surgery in the subsequent year. The nonoperative branch consists of three states: dead, survive - immobile, and survive - mobile. Once patients enter either survive - immobile or survive - mobile, they stay there until they die. (**B**) The surgical branch of extracapsular fractures consists of five health states: dead, well, conversion to arthroplasty, infection revision arthroplasty, and aseptic revision arthroplasty (infection and aseptic revisions are represented by one oval in the figure). The well state includes good and fair outcomes, because distribution and utility data for these separate health states were unavailable for extracapsular fracture. Patients can die, do well, or undergo conversion surgery to arthroplasty during the first year. For patients who had a conversion to arthroplasty, they can die, stay in that state, or have a revision arthroplasty in the subsequent year. The nonoperative branch consists of three states: dead, survive - immobile, and survive - mobile. Once patients enter either survive - immobile or survive - mobile, they stay there until they die.

We estimated the effects of surgical treatment for displaced intracapsular and extracapsular hip fractures through a Markov cohort analysis (Fig. 1). The analysis was limited to patients 65 years and older and results were generated for cohorts ranging in age from 65 to 98 years and summarized using the age distribution of patients with hip fractures who undergo surgery in the United States. Model assumptions (Table 1) were obtained from a systematic literature review, analysis of Medicare claims data, and clinical experts. We recruited and consulted a team of experts with clinical experience in surgical and postoperative care of patients with hip fractures. The expert panel consisted of three orthopaedic surgeons (JOA, RFK, DWL), two physical therapists (HR, JB), and one physical medicine and rehabilitation physician (AK). For assumptions not available in the literature (assumptions related to nonoperative treatment), we relied on the consensus reached by the clinical experts through a series of interviews and questionnaires.

**Table 1:** Clinical parameters and utilities in base Markov model

Clinical parameters/utilities	Displaced intracapsular fractures			Extracapsular fractures		
	Hemiarthroplasty	THA	Nonoperative	Sliding hip screw	Gamma nail	Nonoperative
Clinical parameter						
First-year mortality	3.07 × natural mortality [13]	3.07 × natural mortality [13]	1.33 × 3.07 × natural mortality [13, 18]	3.07 × natural mortality [13]	3.07 × natural mortality [13]	1.33 × 3.07 × natural mortality [13, 18]
Second-year mortality	1.87 × natural mortality [13]	1.87 × natural mortality [13]	1.33 × 1.87 × natural mortality [13, 18]	1.87 × natural mortality [13]	1.87 × natural mortality [13]	1.33 ×1.87 × natural mortality [13, 18]
Rate of conversion to arthroplasty				0.04 [1]	0.06 [1]	NA
Annual rate of revision arthroplasty - aseptic	0.034 [31]	0.0067 [31]	NA	0.0067 [31]	0.0067 [31]	NA
Annual rate of revision arthroplasty - infection	0.0053 [31]	0.0033 [31]	NA	0.0033 [31]	0.0033 [31]	NA
Surgical mortality of revision arthroplasty - aseptic	0.012 [9]	0.012 [9]	NA	0.012 [9]	0.012 [9]	NA
Surgical mortality of revision arthroplasty - infection	0.0193 [9]	0.0193 [9]	NA	0.0193 [9]	0.0193 [9]	NA
Probability of mobility if survive	NA	NA	0.5 [12]	NA	NA	0.5 [12]
Utility						
Dead	0	0	0	0	0	0
Well	0.66 [22]	0.7 [22]	NA	0.54 [10, 27]	0.54 [10, 27]	NA
Conversion to arthroplasty				0.54 [10, 27]	0.54 [10, 27]	NA
Revision arthroplasty - aseptic	0.66 [22]	0.7 [22]	NA	0.54 [10, 27]	0.54 [10, 27]	NA
Revision arthroplasty - infection	0.39 [38, E]	0.39 [38, E]	NA	0.38 [10, E]	0.38 [10, E]	NA
Survive - mobile	NA	NA	0.39 [E]	NA	NA	0.38 [10, E]
Survive - immobile	NA	NA	0 [2]	NA	NA	0 [2]
Disutility - internal fixation				−0.15 [35]	−0.15 [35]	NA
Disutility - initial arthroplasty	−0.15 [35]	−0.15 [35]	NA	−0.15 [35]	−0.15 [35]	NA
Disutility - revision arthroplasty	−0.2 [35]	−0.2 [35]	NA	−0.2 [35]	−0.2 [35]	NA

Numbers in brackets indicate the source of information (ie, reference number of studies cited); E = expert opinion; NA = results not affected by parameter.

We ran separate models for each of four surgical techniques: hemiarthroplasty and THA for displaced intracapsular fractures and intramedullary and extramedullary implants for extracapsular fractures. Internal fixation was not modeled for displaced intracapsular hip fractures since it is not the preferred treatment for older patients [16]. To assess the effect of uncertainty in model assumptions, we conducted a series of sensitivity analyses that tested a reasonable range of all parameters in the Markov model for a representative 80-year-old (Table 2). These sensitivity analyses show the effect of changes in each model parameter (eg, first year mortality rate) on model outputs. We also performed scenario sensitivity analyses in which multiple parameters changed simultaneously to test model outputs under more extreme conditions. The model was estimated in TreeAge Pro 2011 (TreeAge Software, Inc, Williamstown, MA, USA) using the Markov model transition probability matrix. Additional details on the derivation of model assumptions and approach are provided (Appendix 1. Supplemental materials are available with the online version of CORR^®^). Specifically, Appendix 1 includes additional information regarding the use of the clinical experts, the derivation of all-payer payments, and the development of assumptions regarding long-term nursing home use.

**Table 2:** Sensitivity analysis of all model parameters*

Parameter	Value in base model	Value range tested	Range of incremental saving (USD)		Range of incremental QALY	
			Intracapdsular	Extracapsular	Intracapsular	Extracapsular
Rate of long-term nursing home use within immobile patients after nonsurgical treatment	0.9	0.45–1	−16,178 to 109,640	−9704 to 116,113	NA	NA
Rate of long-term nursing home use within surgically treated patients and mobile patients after nonsurgical treatment	0.243/0.481 (age 75–84 years/85+ years)	0.122/0.241–0.365/0.722	136,709–36,539	140,593–45,617	NA	NA
Rate of being mobile after nonsurgical treatment	0.5	0.25–0.75	148,883–24,644	155,356–31,118	3.7–2.4	2.9–1.7
Annual costs of nursing home (USD)	74,498	37,249–111,747	33,850–139,677	37,726–148,747	NA	NA
Annual long-term medical costs after surgical treatment (USD)	12,941	6471–14,790	−128,658–74,790	134,326–81,494	NA	NA
Annual long-term medical costs after nonsurgical treatment (USD)	14,790	12,941–22,185	75,861–130,364	82,335–136,838	NA	NA
Direct medical costs associated with surgical treatment (USD)	Note 1	0.5–1.5 × base value	118,264–55,264	121,835–64,639	NA	NA
Direct medical costs associated with nonsurgical treatment (USD)	Note 2	0.5–1.5 × base value	66,441–107,086	75,982–110,491	NA	NA
One-time home modification cost (USD)	349	175–524	86,821–86,706	93,295–93,180	NA	NA
Ratio of all-cause mortality of nonsurgical group to surgical group in the first 2 years	1.33	1.04–1.71	114,824–51,559	121,297–58,033	3.0–3.2	2.2–2.5
Ratio of all-cause mortality of surgical group to general population in the first 2 years	3.07/1.87 (1st/2nd year)	1.5/1–4.6/2.8	116,597–59,962	125,075–64,529	3.4–2.7	2.6–2.1
Number of years of excess mortality (compared with general population) persists (Note 3)	2	3–10	84,438–75,845	90,459–79,906	2.9–2.6	2.3–1.9
Rate of conversion to arthroplasty (extracapsular only)	0.06	0.03–0.09	NA	93,864–92,610	NA	2.3–2.3
Annual rate of revision arthroplasty (aseptic and infection)	Note 4	0.5–1.5 × base value	93,525–81,322	93,412–93,071	3.1–3.0	2.3–2.3
Mortality of revision arthroplasty (aspetic and infection)	0.012/0.0193(aseptic/infection)	0.006/0.0097–0.018/0.029	86,408–87,120	92,567–93,873	3.2–3.2	2.3–2.3
Utility of being immobile after nonsurgical treatment	0	0–0.2	NA	NA	3.1–2.4	2.3–1.7
Utility of being mobile after nonsurgical treatment	0.39/0.38 (intracapsular/extracapsular)	0.2–0.55	NA	NA	3.6–2.6	2.9–1.8
All disutilities associated with surgery	Note 5	0.5–1.5 × base value	NA	NA	3.1–3.0	2.4–2.3
Utility of being well after surgery (intracapsular)	0.66	0.55–0.79	NA	NA	2.3–3.9	NA
Utility of being well after surgery (extracapsular)	0.54	0.38–0.66	NA	NA	NA	1.3–3.1
Utility after infection revision	0.39/0.38 (intracapsular/extracapsular)	0.2–0.55	NA	NA	3.0–3.1	2.3–2.3

* Sensitivity analysis was performed for an 80-year-old patient receiving either hemiarthroplasty for intracapsular fracture or internal fixation with A Gamma nail for extracapsular fracture. Total savings are net savings in direct medical costs, long-term medical costs, nursing home costs, and home modification costs from surgical treatment relative to nonsurgical treatment. Value range tested was determined either based on literature or 50% higher and lower than value in base model when reasonable range was not available in literature. In some cases, the upper limit or lower limit or both were capped at a certain value to avoid unreasonable parameter assumptions. Note 1 = including direct medical costs of hemiarthroplasty (USD 52,126), THA (USD 49,207), internal fixation (USD 54,054), and revision arthroplasty (USD 44,784); Note 2 = direct medical costs associated with nonsurgical treatment are USD 40,795 for intracapsular and USD 34,509 for extracapsular fractures under the base scenario; Note 3 = for both groups, the annual mortality beyond the second year was set at 1.7 × natural mortality, based on published data [13]; Note 4 = 0.034/0.0053 (aseptic/infection) after hemiarthroplasty and 0.0067/0.0033 (aseptic/infection) after conversion to arthroplasty from internal fixation; Note 5 = including disutility associated with initial hemiarthroplasty/THA and internal fixation (−0.15) and revision arthroplasty (−0.2); QALY = quality-adjusted life year; NA = results not affected by change in parameter.

### Clinical Parameters

Mortality rates after hip fracture vary [11, 20, 22, 29, 33, 39, 40]; we assumed overall relative risks of 3.07 and 1.87 during the first and second years after a hip fracture, with a subsequent return to age-specific levels [13]. These values then were applied to the age-specific natural mortality in the US life tables [4]. For revision, we used the surgical mortality rate of infection revision (1.2%) and aseptic revision (1.9%) reported by Chang et al. [9] for hemiarthroplasty and THA.

Few studies report mortality rates for nonoperative treatment of hip fractures [14, 18, 19, 28]. We used the existing literature to set the mortality rate equal to 1.33 times the mortality rate of patients treated surgically [18]. After the second year, we used the age-specific natural mortality level for surgical and nonsurgical groups.

The long-term revision rate reported by Ravikumar and Marsh [31] was used to estimate the rates for septic and aseptic revision for intracapsular fracture.

For revision of extracapsular fracture treatment, we used first-year revision rates as reported by Adam et al. [1], of 4% and 6% for patients treated with sliding hip screws and intramedullary nails, respectively. We assumed that all patients who needed a reoperation to treat failed internal fixation received a conversion to THA within the first year. For revision rates in subsequent years, we used the intracapsular revision rate that reflects revisions after THA. For both types of hip fractures, we allowed no more than one revision arthroplasty.

The utility values for the health states represented in the model were attained from a review of the literature. The utility values are used to estimate the Quality-Adjusted Life Years (QALYs), which is a standard metric to measure patient quality of life. QALY is the product of the number of additional years to live as a result of a treatment and the utility of the patient health status in those years, with utility usually ranging from 0 for death to 1 for perfect health. For example, if a treatment extends a patient’s life by 2 years and the utility of patient health status after treatment is 0.8, we would say that treatment leads to an additional 1.6 (ie, 2*0.8) QALYs. For the intracapsular and extracapsular models, we used a one-time disutility of −0.15 for arthroplasty and internal fixation and −0.2 for revision arthroplasty [35].

Based on the available literature, we assumed that 50% of the patients who are treated nonoperatively for displaced intracapsular and extracapsular fractures can walk at the conclusion of treatment. For both types of fractures, we set a utility level of 0 for survivors of nonsurgical treatment who cannot walk, based on our estimate using the US valuation of EQ-5D™ [2]. For patients who can mobilize after treatment, their utility was set to be the same as the utility after infection revision based on expert opinion.

### Nursing Home Utilization

We developed estimates of the probability of being in a nursing home before and after a hip fracture using findings from the literature [6, 37], the 2004 National Nursing Home Survey [21], and clinical expert input. The following assumptions were made regarding nursing home use for patients with hip fractures: (1) rates of long-term nursing home use for patients treated surgically and for patients who obtain mobility after nonsurgical treatment of a hip fracture are 16% for patients 65 to 74 years old, 24% for patients 75 to 84 years old, and 48% for patients older than 85 years; and (2) rates of long-term nursing home use for patients treated nonsurgically and immobile are 53% for patients 65 to 74 years old, 57% for patients 75 to 84 years old, and 69% for patients older than 85 years (Appendix 1. Supplemental material is available with the online version of CORR^®^).

### Cost Estimates

Estimates of the direct medical costs (which include inpatient, postacute care, rehabilitation, outpatient, and physician services, but exclude long-term nursing home costs) associated with surgical and nonsurgical treatment (Table 3) were derived from a payer perspective and based on claims for a 5% sample of Medicare beneficiaries in 2009 but adjusted to reflect all payer costs. We accumulated medical costs incurred from hospitalization for hip fracture to 6 months after the hospital discharge from Medicare claims. Long-term (ie, beyond 6 months) annual medical costs are likely to be higher in the nonoperative group than in the surgical group because of increased functional limitations [14]. Based on literature [7, 8, 14] and expert opinion, we assumed long-term annual healthcare costs of USD 12,941 for surgically treated patients and USD 14,790 for nonoperatively treated patients. We assumed an annual nursing home cost of USD 74,498 (2009 USD) based on the 2011 MetLife Market Survey of Nursing Homes [26] and a one-time home modification cost of USD 349 based on our estimates using the 2010 Health and Retirement Study [15].

**Table 3:** Average direct medical costs for 6 months after hip fractures

Type of fracture	Treatment	Average direct medical cost (USD)*
Intracapsular	Hemiarthroplasty	52,126
Intracapsular	THA	49,207
Intracapsular	Nonsurgical treatment	40,795
Extracapsular	Internal fixation	54,054
Extracapsular	Nonsurgical treatment	34,509
Both	Revision hip arthroplasty	44,784

We analyzed 5% sample of 2009 Medicare inpatient claims; ICD-9 diagnosis codes 820.0x and 820.1x were used to identify patients with intracapsular fractures and 820.2x and 820.3x for extracapsular fractures. The following ICD-9 procedure codes were used to identify relevant procedures: 81.51 (THA), 81.52 (hemiarthroplasty), 79.35 (open reduction and internal fixation), 81.53 and 00.70 to 00.73 (revision hip arthroplasty); *cost estimates were risk-standardized for age, sex, and comorbidities and adjusted to reflect different reimbursement rates across payers (eg, private, Medicare, Medicaid, self-insured, and uninsured); estimates include all medical costs (facility and physician fees) across all care settings (including readmissions to hospital, outpatient, and postacute care facilities) from the index hospitalization to 6 months after discharge from the index hospitalization; all costs are expressed in 2009 USD.

## Results

Are the societal benefits of hip fracture surgery enough to offset the direct medical costs? We estimated that the lifetime benefits to society from surgical treatment of hip fracture more than offset the direct medical costs, with average savings per patient of USD 65,279 and USD 67,964 for displaced intracapsular and extracapsular hip fractures, respectively (Table 4). For displaced intracapsular fractures, the surgical treatment cost USD 19,710 more than nonoperative treatment, which was offset by savings of USD 84,990 from lower long-term medical costs and reduced nursing home use. For extracapsular fractures, the direct medical costs for surgical treatment were USD 22,317 higher than that of nonoperative treatment but were offset by savings of USD 90,281. We estimated that the surgical treatment of hip fractures produced an average increase of 2.5 quality-adjusted life years (QALYs) for patients with intracapsular fractures and 1.9 QALYs for patients with extracapsular fracture (Table 4). Surgical treatment was a dominant treatment strategy for hip fracture when total societal savings were considered because it achieved better quality of life at lower cost.

**Table 4:** Societal savings and additional QALYs from surgical treatment of hip fractures

Age group	Societal savings (Δ USD* surgical relative to nonsurgical)					Δ QALY	ICER (Δ USD*/ΔQALY)
	From direct medical costs	From long-term medical costs	From nursing home costs	From home modification costs	Total savings		
	A	B	C	D	E	F	E/F
Intracapsular fractures							
65–69 years	−28,006	18,811	312,781	−129	303,458	6.1	Dominant
70–79 years	−23,941	8993	176,688	−120	161,620	4.2	Dominant
80–89 years	−18,914	−705	49,635	−93	29,923	2.2	Dominant
90+ years	−14,896	−3336	5905	−73	−12,400	0.8	19,544
Overall	−19,710	1969	83,118	−97	65,279	2.5	Dominant
Extracapsular fractures							
65–69 years	−22,447	21,451	318,329	−129	317,203	4.6	Dominant
70–79 years	−22,380	10,901	181,982	−120	170,383	3.1	Dominant
80–89 years	−22,305	378	53,922	−93	31,902	1.6	Dominant
90+	−22,244	−2892	7675	−73	−17,533	0.6	37,073
Overall	−22,317	3190	87,188	−97	67,964	1.9	Dominant

Values are estimated relative to nonsurgical treatments; Column E is calculated as the sum of columns A through D; negative values in E (negative savings) represent increases in societal costs; savings by age groups were weighted by age distribution of the patient population to reach overall savings; all savings are expressed in 2009 USD; QALY = quality-adjusted life year; ICER = incremental cost-effectiveness ratio, calculated using differences in total costs.

For both types of hip fractures, total societal savings and increased QALYs from surgical treatments varied considerably across age groups (Table 4). For example, surgical treatment for an intracapsular fracture achieved approximately USD 160,000 in societal savings and 4.2 QALYs per patient for patients 70 to 79 years old. For patients older than 90 years, surgical treatment was more expensive although still cost-effective.

Because avoided nursing home costs accounted for most of the savings, the results were most sensitive to changes in the parameters related to nursing home use. For an 80-year-old, the breakeven point for the assumption on the percent of patients with hip fractures who would require long-term nursing home use with nonoperative treatment is 37% to 39%, as compared with 24% for surgical patients. That is, avoided nursing home costs would offset the increased cost of surgery for hip fracture when the probability of requiring nursing home use is 13% to 15% higher with nonoperative treatment than with surgical treatment. For all other parameters, the sensitivity analysis showed that surgery always produced a positive net societal savings even with significant deviations (eg, 50%) from the base model assumptions (Table 2). We also performed scenario sensitivity analyses by changing multiple parameters simultaneously to test the robustness of our estimates under more extreme scenarios (Appendix 1. Supplemental materials are available with the online version of CORR^®^). Overall, our saving estimates were relatively robust to parameter assumptions. The QALY estimates were relatively sensitive to the utility of being well after surgery to treat extracapsular fractures.

Nationally, what are the total lifetime benefits of hip fracture surgery for a cohort of patients and to whom do these benefits accrue? At the national level, our findings suggested societal savings from surgical treatment of hip fractures of USD 16 billion for elderly patients in 2009 (based on 307,538 hospital discharges in the United States [3], 90% occurring in patients 65 years or older [36], ½ being extracapsular fractures [12], and 85% of intracapsular fractures displaced [30]). The lifetime total societal benefits from surgical treatment of hip fractures were approximately USD 21 billion with direct medical costs of USD 5 billion. Almost all the benefits (> 95%) were from avoided nursing home costs, which largely accrued to state Medicaid programs and patients. The remaining benefits came from lower long-term medical spending, which largely accrued to government payers (Medicare and Medicaid). As with our individual-level estimates, the national savings estimates are sensitive to the assumption of nursing home use under the nonoperative treatment scenario. Using the range of assumptions for rate of long-term nursing home use among immobile patients after nonsurgical treatment (Table 2), we estimate that societal benefits at the national level range from USD 2 billion to USD 32 billion, with societal savings ranging from USD −3 billion (USD 2 billion to 5 billion) to USD 27 billion (USD 32 billion to 5 billion). Thus, in our most conservative estimate, surgical treatment of hip fracture yields reduced nursing home costs that offset almost 40% of the additional cost of surgery (0.4 = 2 billion/5 billion).

## Discussion

Surgical treatment is considered standard of care for the majority of patients with hip fractures, but evidence regarding the value of surgical treatment of these fractures from a societal perspective is missing in the current literature. In this study, we used a Markov model to assess cost and benefit from operative treatment. We sought to determine whether: (1) the societal benefits of hip fracture surgery are enough to offset the direct medical costs, and (2) the total lifetime benefits of hip fracture surgery for a cohort of patients and to whom these benefits accrue.

The primary limitations of the study occur because of a lack of high-quality data regarding outcomes for nonsurgical treatment of hip fractures. The assumption regarding nursing home use among immobile patients (ie, 90%) is especially important since most of the societal savings were from avoidance of prolonged use of nursing homes. To develop assumptions regarding outcomes under the nonoperative treatment scenario, we relied on a small number of studies and expert opinion. We similarly developed assumptions regarding number of activities of daily living for patients with hip fractures who do not receive surgery and then relied on literature associating healthcare spending with number of activities of daily living. To address the potential effects of bias on the study conclusions from gaps in the data, we conducted extensive sensitivity analyses, which revealed savings even under assumptions more favorable to nonsurgical treatment. There are two additional potential limitations. First, patients undergoing surgery may be healthier at the outset than patients who are treated nonoperatively; thus, difference in outcomes observed in the literature could be influenced by treatment selection. Second, since the 5% Medicare dataset we used to estimate costs includes few patients younger than 65 years, we cannot necessarily extrapolate the results here to younger patients.

We estimated age-weighted, lifetime savings to society for surgical treatment of hip fractures to be more than USD 65,000 per patient, with surgical treatment of hip fractures producing 2.5 and 1.9 additional QALYs compared with nonsurgical treatment for displaced intracapsular and extracapsular hip fractures, respectively. Almost all of the societal savings from surgical treatment of hip fracture were from avoided nursing home costs. Although there are no comparable studies in the literature showing the value of surgical treatment for hip fractures, studies have documented the high personal and financial cost of hip fracture. Braithwaite et al. [6] estimated that a hip fracture reduces life expectancy by 1.8 years with the lifetime cost of a hip fracture at USD 81,300 (1997 dollars). Approximately 44% of these costs were associated with nursing facility expenses. Other studies have documented substantial reductions in functional status with hip fractures [5, 6, 10, 12, 17, 24, 41]. Consistent with our findings of savings from surgical treatment, studies of nonoperative treatment for hip fractures, while limited, report high rates of immobility (45%) [12] and dependency (29% of surgically treated patients versus 57% nonoperatively treated) [18].

At the national level, our findings suggested substantial societal savings from surgical treatment of hip fractures. We estimated that the lifetime total societal savings from surgical treatment of hip fractures was USD 16 billion for elderly patients treated in 2009, mainly from avoidance of nursing home costs. By comparison, Braithwaite et al. [6] estimated lifetime cost for all hip fractures in the US of at least USD 20 billion. Thus, while hip fractures impose substantial societal costs, the results show that surgery has an important role in minimizing the societal burden of hip fractures.

Our study is the first to our knowledge to quantify the economic value of surgical treatment of hip fractures in terms of reduced lifetime societal costs. The scope of factors considered for the calculation of value extended beyond direct medical treatment costs to the long-term medical costs associated with impaired functioning and to nursing home costs accumulated during a lifetime. Although the magnitude of the societal savings depends on model assumptions, the finding of societal savings is robust to a range of parameter values. Because the study focused on the cohort of patients receiving surgery, our results should not be interpreted as suggesting that surgery is appropriate for all patients with hip fractures. This analysis, however, serves as an important benchmark for the economic value of surgery for hip fractures. The method used in our study can be used in future studies to show the additional economic effects as hip fracture care is improved. Future studies also might consider that additional benefits to society from improved employment and productivity resulted from successful surgical treatment of hip fractures, as more elderly workers choose to stay in the labor force longer [23].

## Electronic supplementary material

Below is the link to the electronic supplementary material.

Supplementary material 1 (DOC 62 kb)
