# Supplementary material for: Surgery for Hip Fracture Yields Societal Benefits That Exceed the Direct Medical Costs
Source: Clin Orthop Relat Res. 2014 Aug 5;472(11):3536–46. doi: 10.1007/s11999-014-3820-6 (PMC4182375; doi:10.1007/s11999-014-3820-6)
Supplement: Supplementary file 1 — Supplementary material 1 (DOC 62 kb) [file 11999_2014_3820_MOESM1_ESM.doc]

**Appendix 1.** The derivation of model assumptions and approach

Consultation of Clinical Experts

To validate the assumptions used in the Markov model, we recruited and consulted a team of experts with considerable clinical experience in surgical and postoperative care of patients with hip fractures. Our team of clinical experts consisted of three orthopaedic surgeons (JA, RK, DL), two physical therapists (JB, HR), and one physician (AK) specializing in physical medicine and rehabilitation. For assumptions not available in the literature (mostly assumptions related to nonoperative treatment), we relied on the consensus reached by the clinical experts. We used sensitivity analysis to test the alternative parameters suggested by the clinical experts. For example, some of the experts believed that the functional outcome is 10% to 20% worse after aseptic revision arthroplasty than after the initial arthroplasty. We tested these alternative parameters in the sensitivity analysis.

Estimating the EQ-5D™ Utility Score for Immobile Survivors of Nonsurgical Treatment

EQ-5D™ is a standardized generic measure of health-related quality of life that is widely used in clinical and economic evaluations of health care. The ED-5D™ measure consists of five questions on the following topics: mobility, self-care, usual activities, pain/discomfort, and anxiety/depression. For each question, a score of 1 indicates no problems, 2 indicates moderate problems, and 3 indicates severe problems. For patients who are immobile after nonoperative treatment, it is reasonable to assign a score of 3 on mobility, self-care, and usual activities. Additionally, we expect these patients to experience at least moderate pain and anxiety/depression (ie, a score of 2 or 3). Based on the US valuation of EQ-5D™ [1], the average utility score of the four scenarios (2 or 3 on pain/discomfort and anxiety/depression questions) is 0.

Nursing Home Utilization

We developed estimates of the probability of being in a nursing home before and after a hip fracture using findings from Sugarman et al. [15] and the 2004 National Nursing Home Survey [12]. Sugarman et al. [15] reported hip fracture incidence rates for the elderly population residing in nursing home and community settings. We multiplied these incidence rates by the estimated number of people residing in nursing homes in 2004 and those in the community. We estimated that a person between 65 and 74 years old who experiences a hip fracture had a 3.7% probability of already being in a nursing home at the time of fracture. This probability increases to 13% for persons 75 to 84 years old and to 40.4% for persons older than 85 years.

We made two assumptions to estimate the rate of long-term nursing home use after hip fracture among patients with surgery or mobile patients after nonoperative treatment. First, 13% of community-dwelling patients before hip fracture will need long-term use of a nursing facility after the hip fracture. This rate was reported by Braithwaite et al. [4], who reviewed US reports on long-term nursing home use after hip fractures among previous community dwellers. Second, all patients who resided in nursing homes before their fractures were assumed to stay in nursing homes after treatment. Based on these two assumptions, the estimated rates of long-term nursing home use for patients treated surgically and for patients who obtain mobility after nonsurgical treatment of hip fracture are 16.2% for patients 65 to 74 years old, 24.3% for patients 75 to 84 years old, and 48.1% for patients older 85 years. For immobile patients treated nonoperatively, we assumed 90% of them needed long-term use of a nursing home based on expert opinion.

The following is an example of our calculation of long-term nursing home use for patients 85 years or older. Suppose we have 100 patients 85 years or older undergoing treatment for hip fracture. Based on Sugarman et al. [15], 40 would be expected to reside in a nursing home before the hip fracture, while 60 would reside in the community. Based on Braithwaite et al. [4], after surgical treatment of hip fractures, we assumed that 48 would reside in a nursing home; this includes the 40 who were originally in a nursing home plus an additional eight (13% x 60) who previously were in the community.

With nonsurgical treatment, we assumed that 90% of immobile patients would be in a nursing home after hip fracture; this includes the original nursing home residents. Moreover, we assume the percentage of mobile patients treated nonsurgically and in a nursing home after the fracture would be equal to the percentage treated surgically. Based on Handoll and Parker [10], we assumed that 50% of patients with hip fracture treated nonsurgically would be mobile and 50% would be immobile. Thus, the number of nonsurgically treated patients with hip fractures residing in a nursing home after fracture would be:

Total 100 nonsurgical treated (85+ years old)

Immobile after fracture = 50

Number of immobile patients using nursing home after fracture: 45 = 50 x 0.90

Mobile patients after fracture = 50

Number of mobile patients using nursing home after fracture: 24 = 50 x (0.40 + [0.13 x 0.60])

Total nonsurgically treated patients in nursing home after fracture: 69 = 45 + 24

As shown above, for those 85 years and older, we assumed that 48% of patients with surgically repaired hip fractures would be in a nursing home after the hip fracture as compared with 69% if treated nonsurgically. Similar calculations were performed to estimate nursing home use for patients 65 to 74 years old and for patients 75 to 84 years old.

ICD-9 Diagnosis and Procedure Codes Used for Cost Estimates

We used ICD-9 diagnosis codes 820.0x and 820.1x to identify patients admitted for intracapsular fractures and 820.2x and 820.3x for extracapsular fractures. The following ICD-9 procedure codes were used to identify the relevant surgical techniques: 81.51 for THA, 81.52 for hemiarthroplasty, 79.35 for open reduction and internal fixation, and 81.53 and 00.70 to 00.73 for revision hip arthroplasty.

Cost Estimates

Estimates of the direct medical costs associated with surgical and nonsurgical treatment were based on our analysis of a 5% sample of Medicare claims data in 2009. We used ICD-9 diagnosis and procedure codes to identify patients admitted for intracapsular and extracapsular fractures and surgically treated. The direct medical costs reflect all-payer payments for inpatient, outpatient, physician, and postacute care (eg, skilled nursing facilities, home health services). We accumulated medical costs incurred from the day patients were hospitalized for hip fracture to 6 months after the index hospital discharge. We risk-standardized costs using a regression model and controlled for age, sex, and comorbidities using the Elixhauser comorbidity index [2, 7] created using the secondary diagnosis codes of the index hospitalization.

The long-term (ie, beyond the first 6 months) annual medical costs are likely to be higher in the nonoperative group than in the surgical group because patients treated nonoperatively experience more functional limitations after treatment [10]. Chan et al. [6] estimated the average risk-standardized cost ratio by the number of restrictions in Activities of Daily Living (ADLs) in the Medicare population. They reported annual healthcare costs of Medicare beneficiaries with one to two, three to four, and five to six ADL restrictions were 40%, 60%, and 130% higher than those with zero ADL restrictions in 1997, respectively. Bentler et al. [3] reported the average number of ADL restrictions is in the range of one to two at 2 years after hip fractures. For patients treated nonoperatively, we assumed the number of ADL restrictions after treatment lay within the range of three to four, based on expert opinion. Based on data reported by Chan et al. [6] and updated to 2009 USD [5], we estimated long-term annual healthcare costs of USD 12,941 for surgically treated patients and USD 14,790 for nonoperatively treated patients. Throughout this study, medical costs refer to payments made by third-party payers.

Our estimates of annual nursing home costs rely on the 2011 MetLife Market Survey of Nursing Homes [14], which reported the national average rate for a semiprivate room at a nursing home was USD 78,110 in 2011 (ie, USD 74,498 in 2009 since all costs are expressed in 2009 USD in this analysis). We set a one-time home modification cost of USD 349 for patients returning to the community after treatment, based on our estimates using the 2010 Health and Retirement Study [11].

Converting Medicare Costs to All-payer Costs

Cost estimates based on Medicare payment rates may underestimate payments made by private insurers and overestimate payments made by Medicaid, self-insured, and uninsured patients. To reconcile these differences, we adjusted our estimates of direct medical costs using payment rates of other insurers (as a percentage of the Medicare rate) and then weighted by the national distribution of payers for treatment of hip fractures. We set the payment rate of Medicaid and self-pay patients as 80% and 50% of the Medicare rate, respectively. For private insurers, we used the payment rates reported in the literature. Ginsburg [9] estimated that private insurers, on average, paid 139% of the Medicare payment rates for inpatient care nationally in 2008. He also reported private insurer payments as a percentage of Medicare rates for outpatient services in selected areas, ranging from 193% in Cleveland, OH, USA, to 368% in San Francisco, CA, USA. We used the median of the reported range, which is 280%, to adjust costs of outpatient services. The Medicare Payment Advisory Committee [13] estimated that the private rate for physician services was, on average, 123% of the Medicare rate across all services and areas in 2003. For all other patients, including those paid by Workers Compensation, we assumed their rate was the same as the average rates of Medicare and private insurers.

Estimating Home Modification Costs After Hip Fracture

We used the Health and Retirement Study 2010 early release public use dataset [11], a longitudinal panel study that surveys older Americans every 2 years, to estimate the home modification costs after hip fracture. The Health and Retirement Study asked the respondents whether they had modified their home to make it easier for an older or disabled person to live. Respondents who modified their homes then were asked about the out-of-pocket expense for the home modification. This expense is likely to reflect the total cost of home modification since only 6% of the elderly people who modified their homes reported that insurance or governmental programs covered some of the costs [8]. We identified people older than 65 years who did not have a hip fracture in the previous survey (eg, Health and Retirement Study 2008) but experienced a hip fracture between the previous and the current survey and lived in the community after the fracture. In this population, we estimated the average out-of-pocket expense of home modification to be USD 349. For respondents who experienced a hip fracture in the previous survey but not in the current survey, the average out-of-pocket expense of home modification was USD 23, which suggests that long-term cost of home modification after hip fracture is trivial. Therefore, in our model, we set a one-time home modification cost of USD 349 for patients living in the community after hip fracture.

Details on Scenario Sensitivity Analyses for Intracapsular Fractures

We performed scenario sensitivity analyses, where the three parameters with the largest effect on savings were varied simultaneously, to test the robustness of our estimates under improbable scenarios. When we (1) decreased the nursing home use rate among immobile patients after nonsurgical treatment to 45% (a 50% reduction), (2) increased the rate of being mobile after nonsurgical treatment to 75% (a 50% increase), and (3) increased the nursing home use rate by 50% (capped at 45%) for patients treated surgically and mobile patients treated nonsurgically (all three parameter changes work together to reduce the saving estimate), the total societal saving would be a negative USD 34,477, which means surgery produced an additional cost of USD 12,122 in addition to a higher direct medical cost.

When the three values worked together in the direction to increase savings with a 50% deviation, the total societal savings was a little more than USD 250,000. When we changed the deviation from 50% to 25%, the indirect savings from surgery offset 89% of higher direct medical costs (ie, a net saving of negative USD 2480) when the three parameters worked together to reduce savings. When they worked together to increase savings, the net societal saving was USD 175,073. Scenario sensitivity analyses on extracapsular fractures produced similar results.

**References**

Agency for Healthcare Research and Quality. Calculating the U.S. population-based EQ-5D™ index score. Available at: http://www.ahrq.gov/rice/EQ5Dscore.htm. Accessed April 23, 2012.

Agency for Healthcare Research and Quality. Comorbidity software, Version 3.7. Available at: http://www.hcup-us.ahrq.gov/toolssoftware/comorbidity/comorbidity.jsp. Accessed July 20, 2012.

# Bentler S, Liu L, Obrizan M, Cook EA, Wright KB, Geweke JF, Chrischilles EA, Pavlik CE, Wallace RB, Ohsfeldt RL, Jones MP, Rosenthal GE, Wolinsky FD. The aftermath of hip fracture: discharge placement, functional status change, and mortality. *Am J Epidemiol*. 2009;170:1290-1299.

[Braithwaite RS](http://www.ncbi.nlm.nih.gov/pubmed?term=Braithwaite RS%5BAuthor%5D&cauthor=true&cauthor_uid=12588580), [Col NF](http://www.ncbi.nlm.nih.gov/pubmed?term=Col NF%5BAuthor%5D&cauthor=true&cauthor_uid=12588580), [Wong JB](http://www.ncbi.nlm.nih.gov/pubmed?term=Wong JB%5BAuthor%5D&cauthor=true&cauthor_uid=12588580). Estimating hip fracture morbidity, mortality and costs. [*J Am Geriatr Soc.*](http://www.ncbi.nlm.nih.gov/pubmed/12588580)2003;51:364-370.

Centers for Medicare and Medicaid Services. Market basket data. Available at: http://www.cms.gov/Research-Statistics-Data-and-Systems/Statistics-Trends-and-Reports/MedicareProgramRatesStats/MarketBasketData.html. Accessed July 20, 2012.

Chan L, Beaver S, MacLehose RF, Jha A, Maciejewski M, Doctor JN. Disability and health care costs in the Medicare population. *Arch Phys Med Rehabil.* 2002;83:1196-1201.

[Elixhauser A](http://www.ncbi.nlm.nih.gov/pubmed?term=Elixhauser A%5BAuthor%5D&cauthor=true&cauthor_uid=9431328), [Steiner C](http://www.ncbi.nlm.nih.gov/pubmed?term=Steiner C%5BAuthor%5D&cauthor=true&cauthor_uid=9431328), [Harris DR](http://www.ncbi.nlm.nih.gov/pubmed?term=Harris DR%5BAuthor%5D&cauthor=true&cauthor_uid=9431328), [Coffey RM](http://www.ncbi.nlm.nih.gov/pubmed?term=Coffey RM%5BAuthor%5D&cauthor=true&cauthor_uid=9431328). Comorbidity measures for use with administrative data. [*Med Care.*](http://www.ncbi.nlm.nih.gov/pubmed/9431328?dopt=Abstract)1998;36:8-27.

1. Freedman VA, Agree EM. Home modifications: use, cost, and interactions with functioning among near-elderly and older adults. US Department of Health and Human Services. Available at: http://aspe.hhs.gov/daltcp/reports/2008/homemod.pdf. Accessed August 17, 2012.

Ginsburg PB. Wide variation in hospital and physician payment rates evidence of provider market power. *Res Brief*. 2010;16:1-11.

1. Handoll HH, Parker MJ. Conservative versus operative treatment for hip fractures in adults. *Cochrane Database Syst Rev.* 2008;3:CD000337.

# Health and Retirement Study, 2010 Final Release public use dataset. Produced and distributed by the University of Michigan with funding from the National Institute on Aging (grant number NIA U01AG009740). Ann Arbor, MI.

# Jones AL, Dwyer LL, Bercovitz AR, Strahan GW. The National Nursing Home Survey: 2004 Overview. Vital Health Statistics. National Center for Health Statistics, Hyattsville, MD. Available at: <http://www.cdc.gov/nchs/data/series/sr_13/sr13_167.pdf>. Accessed May 27, 2014.

# Medicare Payment Advisory Committee. *Report to the Congress: Medicare Payment Policy.* Washington DC: Medicare Payment Advisory Committee; March 2005. Available at: <http://www.medpac.gov/documents/june05_entire_report.pdf>. Accessed July 10, 2014.

# MetLife Mature Market Institute. Market Survey of Long-Term Care Costs. The 2011 MetLife Market Survey of Nursing Home, Assisted Living, Adult Day Services, and Home Care Costs. New York, NY: MetLife Mature Market Institute; October 2011. Available at: <https://www.metlife.com/assets/cao/mmi/publications/studies/2011/mmi-market-survey-nursing-home-assisted-living-adult-day-services-costs.pdf>. Accessed July 10, 2010.

1. [Sugarman JR](http://www.ncbi.nlm.nih.gov/pubmed?term=Sugarman JR%5BAuthor%5D&cauthor=true&cauthor_uid=12366616), [Connell FA](http://www.ncbi.nlm.nih.gov/pubmed?term=Connell FA%5BAuthor%5D&cauthor=true&cauthor_uid=12366616), [Hansen A](http://www.ncbi.nlm.nih.gov/pubmed?term=Hansen A%5BAuthor%5D&cauthor=true&cauthor_uid=12366616), [Helgerson SD](http://www.ncbi.nlm.nih.gov/pubmed?term=Helgerson SD%5BAuthor%5D&cauthor=true&cauthor_uid=12366616), [Jessup MC](http://www.ncbi.nlm.nih.gov/pubmed?term=Jessup MC%5BAuthor%5D&cauthor=true&cauthor_uid=12366616), [Lee H](http://www.ncbi.nlm.nih.gov/pubmed?term=Lee H%5BAuthor%5D&cauthor=true&cauthor_uid=12366616). Hip fracture incidence in nursing home residents and community-dwelling older people, Washington State, 1993-1995. [*J Am Geriatr Soc.*](http://www.ncbi.nlm.nih.gov/pubmed/12366616) 2002;50:1638-1643.
